# Supplementary figures and images for: Phospho-regulation of the Shugoshin - Condensin interaction at the centromere in budding yeast
Source: PLoS Genet. 2020 Aug 18;16(8):e1008569. doi: 10.1371/journal.pgen.1008569 (PMC7454948; doi:10.1371/journal.pgen.1008569)

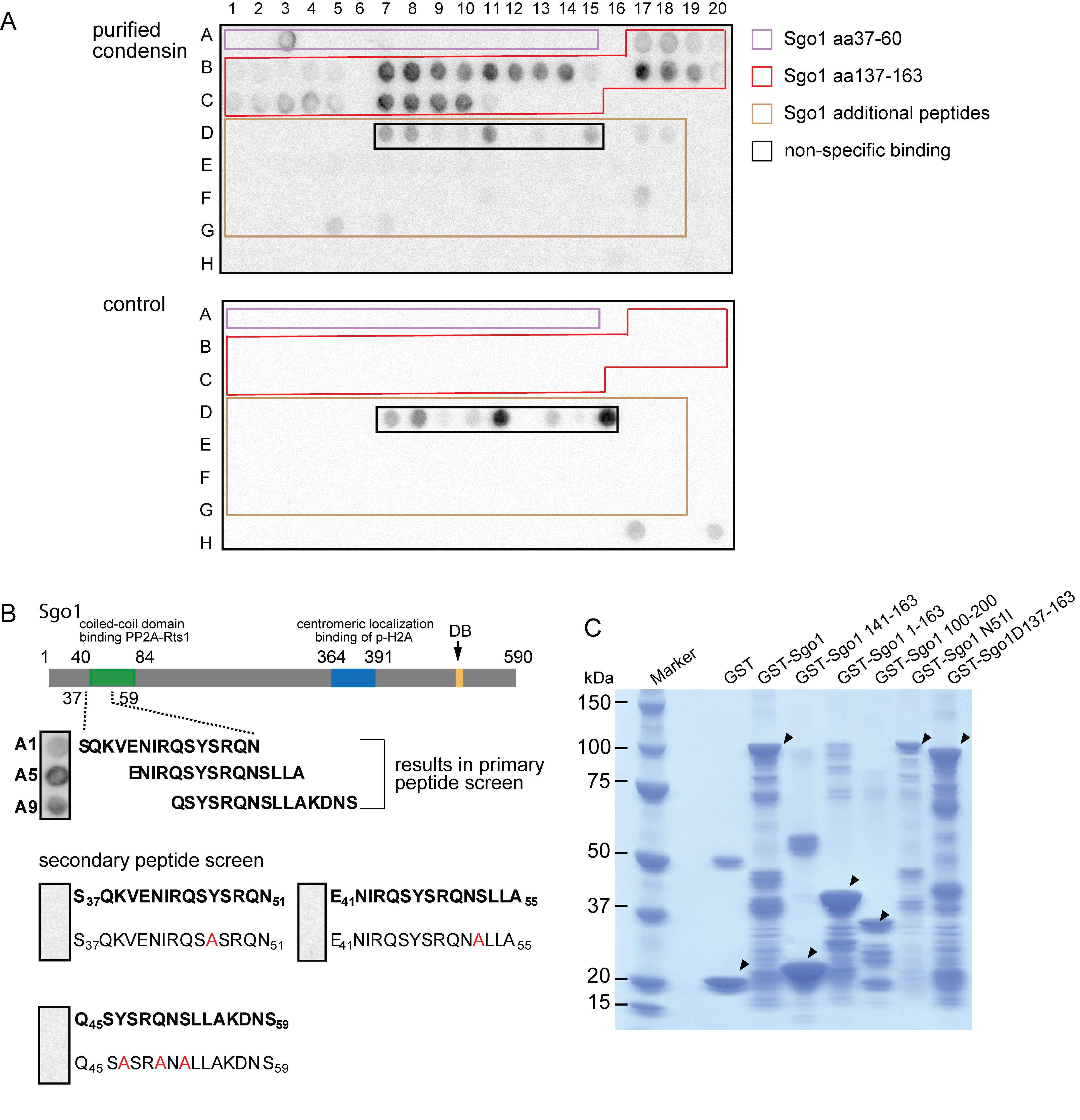

Supplement: S1 Fig — (A) Far western was performed on a peptide print of Sgo1 using tagged condensin purified from budding yeasts (upper blot) or untagged control. The nonspecific binding is marked in black. Positions A1-A15: peptides covering the Sgo1 sequence from aa 37 to 60 (A9-A13 in the primary screen) and its various mutations. Positions A17- C15: peptides covering the Sgo1 sequence from aa 137 to aa 167 and its various mutations (B15 -B20 in the primary screen). D1 to G20: additional individual peptides that showed week positivity in the primary screen. Positions H17—H20: tags for antibody controls. (B) Comparison of the primary and secondary peptide plot of the putative interacting region aa 37–60. Upper blot—primary screen, lower blot—secondary screen. Several mutant peptides were added to evaluate the specificity of the putative binding. (C) GST-tagged Sgo1 purified from E. coli, wt and the mutant variants. (TIF) [file pgen.1008569.s001.tif]

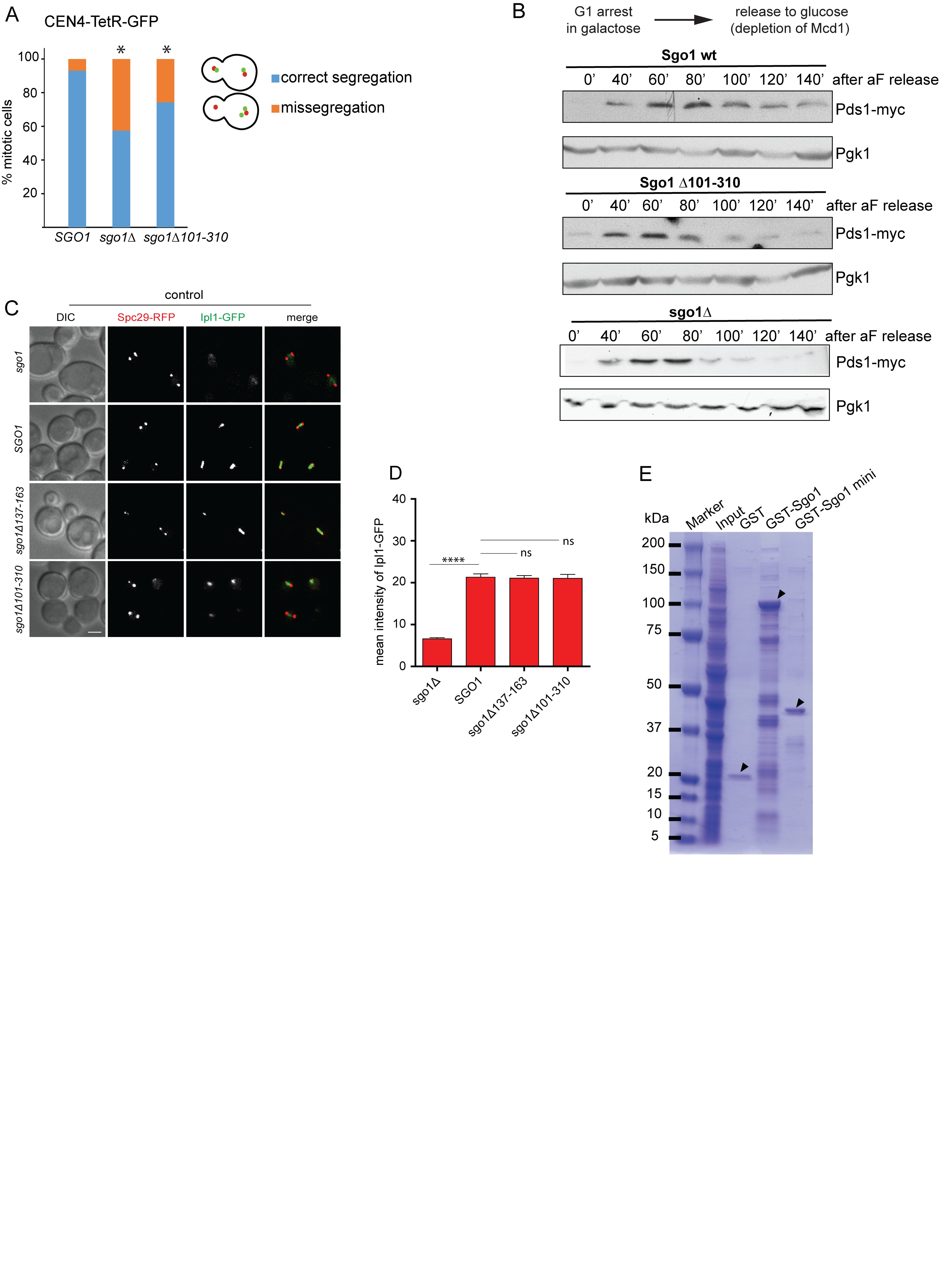

Supplement: S2 Fig — (A) Chromosome segregation (centromeres of chromosome 4 labeled by tetO/TetR-GFP) in cells released from an arrest in medium containing nocodazole (2 h treatment). Cells were collected 75 min after the release from nocodazole, fixed and imaged. (B) Cell cycle arrest in response to loss of sister chromatid cohesion monitored by immunoblotting of Pds1-9myc degradation in cell lysates of wild type SGO1, sgo1Δ101–310 and sgo1Δ. Cells carrying Gal10-Mcd1 allele were arrested in G1 by α-factor and released into medium with and without glucose. Samples were collected at indicated time points. Pgk1 –phosphoglycerate kinase (loading control). (C) Examples of Ipl1-GFP localization in sgo1Δ, wild type SGO1, sgo1Δ137–163 and sgo1Δ101–310. (D) Quantification of Ipl1-GFP mean intensity for control condition in C. (E) Purified GST-tagged Sgo1 wild type and Sgo1-mini. Unpaired t test was used for statistical analysis in D. ****, p<0.0001; ns (not significant). (TIF) [file pgen.1008569.s002.tif]

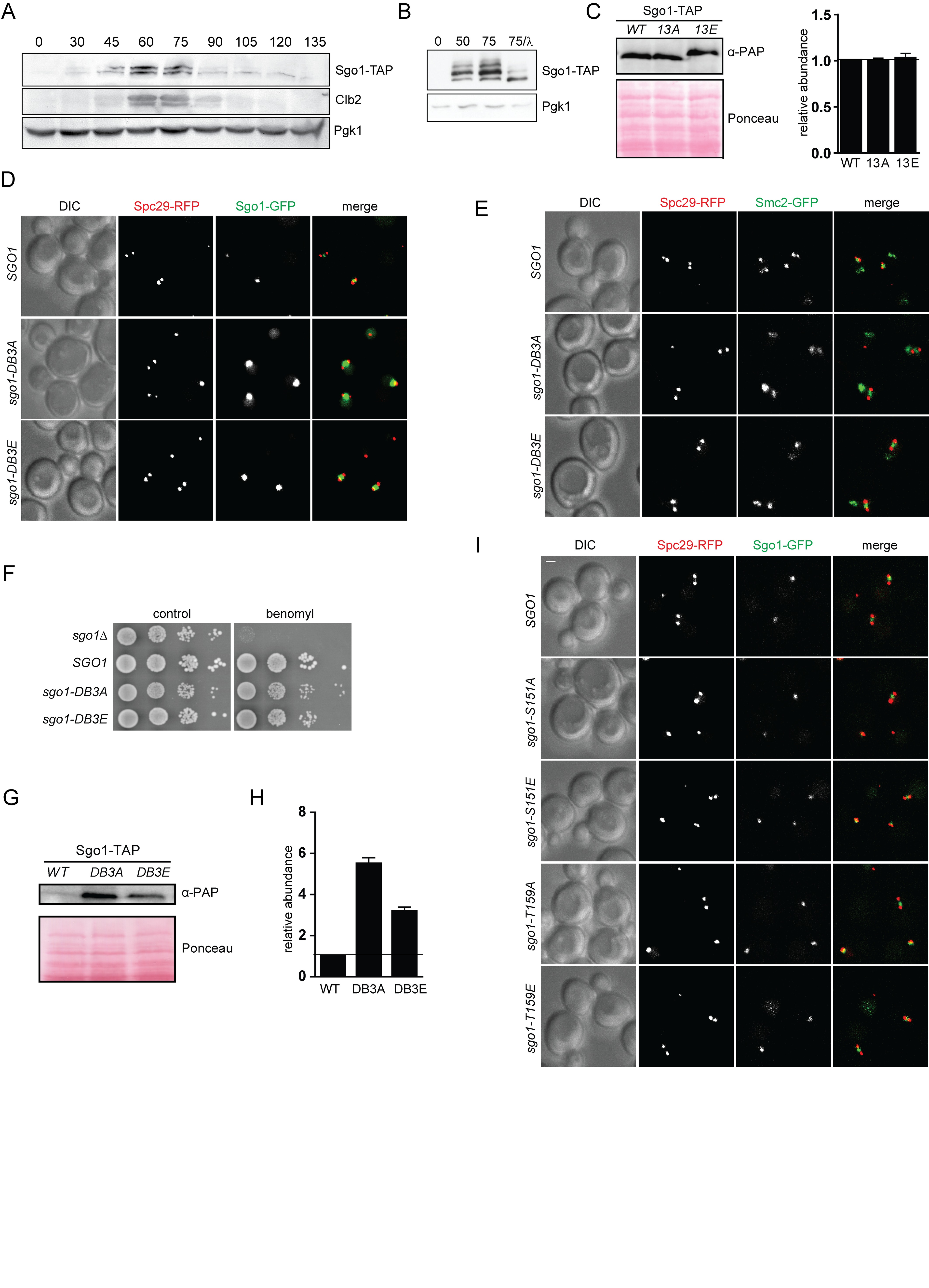

Supplement: S3 Fig — (A) Migration of Sgo1-TAP in Phostag-PAGE. Cells were collected at specified time points after a release from a G1 arrest by α-factor. After 45 min, α-factor was again added to limit the cells to one cell cycle. (B) Migration of Sgo1-TAP in Phostag-PAGE after release from α-factor. The sample 75/l was treated with l-phosphatase for 60 min. (C) Levels of phosphomimic (13E) and phosphoresistant (13A) mutants of Sgo1—immunoblot and the quantification of three biological replicates. (D) Localization of Sgo1-GFP-DB3A and DB3E to preanaphase spindle. (E) Localization of condensin in Sgo1-DB3A and Sgo1-DB3E mutants. (F) Sensitivity of sgo1-DB3A and sgo1-DB3E mutants to benomyl. (G, H) Protein levels of Sgo1-DB3A and Sgo1-DB3E - immunoblot and quantification. (I) Localization of the Sgo1-T159 and Sgo1-S151 mutants to the preanaphase spindle. (TIF) [file pgen.1008569.s003.tif]

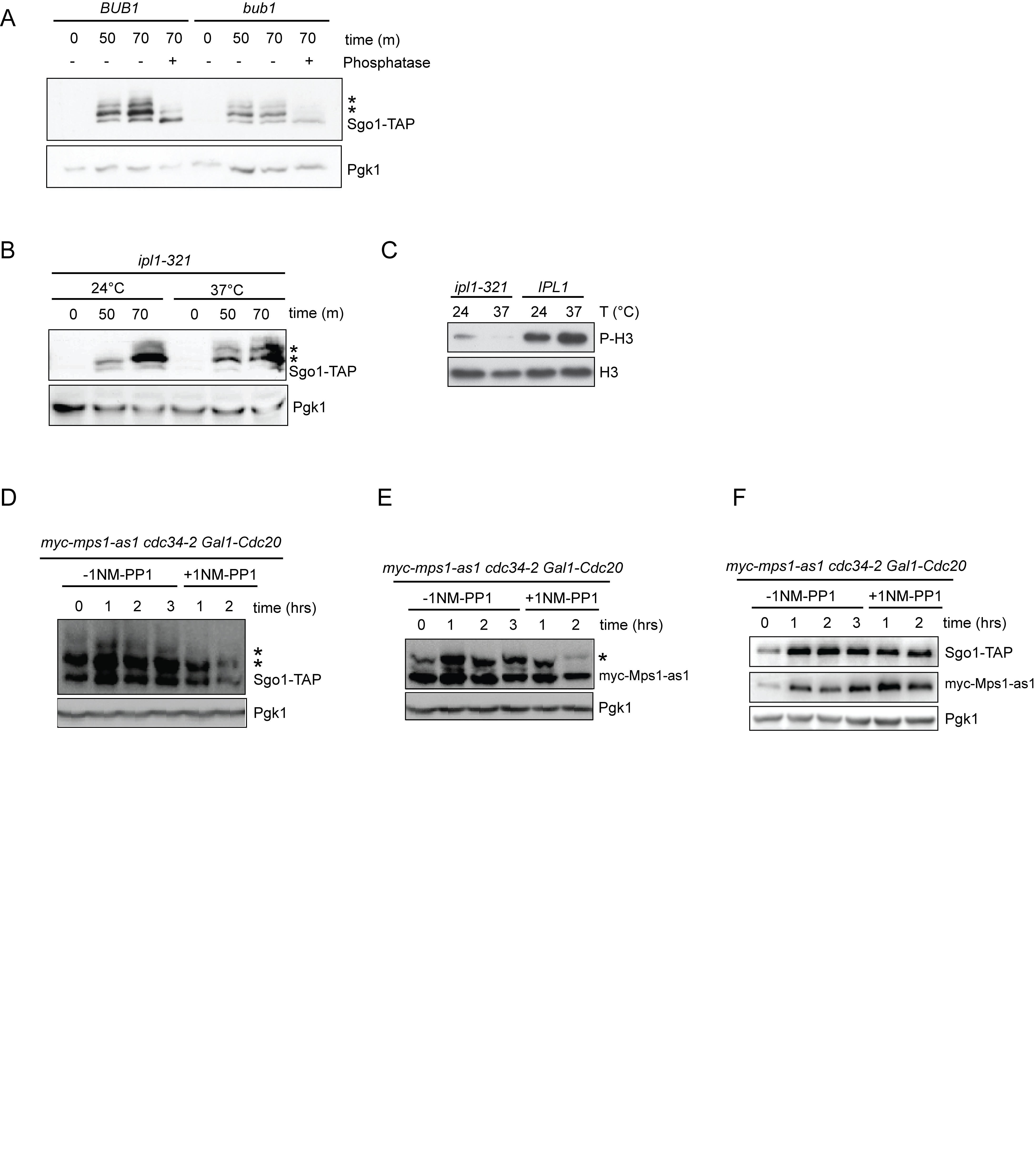

Supplement: S4 Fig — (A) Migration of Sgo1-TAP in Phostag-PAGE after release from α-factor showed similar pattern in the presence or absence of Bub1. (B) Migration of Sgo1 in Phostag-PAGE was not affected by the presence or absence of Ipl1 kinase activity; (C) in contrast, H3 phosphorylation was almost completely abolished in cells expressing ipl1-321, a temperature-sensitive mutant that impairs the Ipl1 kinase activity at 37°C. (D) Migration of Sgo1-TAP extracted from cells harboring an analog sensitive allele of MPS1 (mps1-as1) in Phostag-PAGE in presence or absence of the ATP analog 1NM-PP1. (E) Autophosphorylation of Mps1 was monitored in the same experiment as an internal control for kinase activity. (F) Immunoblots showing that total protein level of Sgo1, mps1-as1 and Pgk1 were not affected by the prolonged 1NM-PP1 treatment. * marks the phosphorylated form of the indicated proteins. (TIF) [file pgen.1008569.s004.tif]
