# Supplementary material for: Phospho-regulation of the Shugoshin - Condensin interaction at the centromere in budding yeast
Source: PLoS Genet. 2020 Aug 18;16(8):e1008569. doi: 10.1371/journal.pgen.1008569 (PMC7454948; doi:10.1371/journal.pgen.1008569)
Supplement: S1 Table — (DOCX) [file pgen.1008569.s005.docx]

Supplementary Table 1. Yeast strains used and created in this study

All listed strains are derived from W303 *MATa*, except *s288c* strains. ade2-1: ochre mutation;

can1-100: ochre mutation; ura3-1: mutation causing uracil auxotrophy; leu2-3, 112: mutation causing lysine auxotrophy; trp1-1: mutation causing tryptophan auxotrophy; his3-11: mutation causing histidine auxotrophy.

| **Strain number** | ***S. cerevisiae* Strain** | **Relevant Genotype** | **Source** |
| --- | --- | --- | --- |
| YZ1359 | *SGO1*-TAP *P_GAL1_*-*cik1*-*CC*-TAP | *MATa ade2-1 can1-100 his3-11 leu2-3,112 trp1-1 ura3-1 PSGO1::pRS405-PSGO1-SGO1-TAP::LEU2 sgo1::hphNT1 ura3-1::pRS406-PGAL1-cik1-CC-TAP::URA3* | Andreas Wallek |
| YZ1360 | *sgo1∆ P_GAL1_*-*cik1*-*CC*-TAP | *MATa ade2-1 can1-100 his3-11 leu2-3,112 trp1-1 ura3-1 sgo1::hphNT1 ura3-1::pRS406-PGAL1-cik1-CC-TAP::URA3* | Andreas Wallek |
| YZ1634 | W303 Smc2-6HA Rts1-9myc Ycg1-3FLAG | *MATa ade2-1 can1-100 his3-11 leu2-3,112 trp1-1 ura3-1 tSMC2::6HA-HIS3 tRTS1::9myc-TRP1 TYcg1::3FLAG-NatN* | Yehui Wu |
| YZ1670 | W303 sgo1∆ pRS405-PSGO1-Sgo1 S151A-TAP | *MATa ade2-1 can1-100 his3-11 leu2-3,112 trp1-1 ura3-1 SGO1::hphNT1 sgo1-S151A-TAP-LEU2* | This work |
| YZ1674 | W303 sgo1∆ pRS405-PSGO1-Sgo1 S148 A S151A-TAP | *MATa ade2-1 can1-100 his3-11 leu2-3,112 trp1-1 ura3-1 SGO1::hphNT1 sgo1-S148A-S151A-TAP-LEU2* | This work |
| YZ1675 | W303 sgo1∆ pRS405-PSGO1-Sgo1 S148 A S151A T159A-TAP | *MATa ade2-1 can1-100 his3-11 leu2-3,112 trp1-1 ura3-1 SGO1::hphNT1 sgo1-S148A-S151A-T159A-TAP-LEU2* | This work |
| YZ1676 | W303 sgo1∆ Rts1-eGFP Spc29-RFP pRS405-PSGO1-Sgo1 S148 A S151A-TAP | *MATa ade2-1 can1-100 his3-11 leu2-3,112 trp1-1 ura3-1 SGO1::hphNT1 tRTS1::eGFP-HIS3 tSPC29-RFP-natNT2 sgo1-S148A-S151A-TAP-LEU2* | This work |
| YZ1677 | W303 sgo1∆ Rts1-eGFP Spc29-RFP pRS405-PSGO1-Sgo1 S148 A S151A T159A-TAP | *MATa ade2-1 can1-100 his3-11 leu2-3,112 trp1-1 ura3-1 SGO1::hphNT1 tRTS1::eGFP-HIS3 tSPC29-RFP-natNT2 sgo1-S148A-S151A-T159A-TAP-LEU2* | This work |
| YZ1678 | W303 sgo1∆ Rts1-eGFP Spc29-RFP pRS405-PSGO1-Sgo1 S151A-TAP | *MATa ade2-1 can1-100 his3-11 leu2-3,112 trp1-1 ura3-1 SGO1::hphNT1 tRTS1::eGFP-HIS3 tSPC29-RFP-natNT2 sgo1-S151A-TAP-LEU2* | This work |
| YZ1679 | W303 sgo1∆ Spc29-RFP pRS405-PSGO1-Sgo1 S148 A S151A-GFP | *MATa ade2-1 can1-100 his3-11 leu2-3,112 trp1-1 ura3-1 SGO1::hphNT1 tSPC29::RFP-kanMX sgo1-S148A-S151A-GFP-LEU2* | This work |
| YZ1680 | W303 sgo1∆ Spc29-RFP pRS405-PSGO1-Sgo1 S148 A S151A T159A-GFP | *MATa ade2-1 can1-100 his3-11 leu2-3,112 trp1-1 ura3-1 SGO1::hphNT1 tSPC29::RFP-kanMX sgo1-S148A-S151A-T159A-GFP-LEU2* | This work |
| YZ1681 | W303 sgo1∆ Spc29-RFP pRS405-PSGO1-Sgo1 S151A-GFP | *MATa ade2-1 can1-100 his3-11 leu2-3,112 trp1-1 ura3-1 SGO1::hphNT1 tSPC29::RFP-kanMX sgo1-S151A-GFP-LEU2* | This work |
| YZ1687 | W303 sgo1∆ pRS405-PSGO1-Sgo1 mini-NLS-TAP | *MATa ade2-1 can1-100 his3-11 leu2-3,112 trp1-1 ura3-1 SGO1::hphNT1 sgo1-mini-SV40NLS-TAP-LEU2* | This work |
| YZ1688 | W303 sgo1∆ pRS405-PSGO1-NLS-Sgo1 mini-TAP | *MATa ade2-1 can1-100 his3-11 leu2-3,112 trp1-1 ura3-1 SGO1::hphNT1 SV40NLS-sgo1-mini-TAP-LEU2* | This work |
| YZ1689 | W303 sgo1∆ Rts1-eGFP Spc29-RFP pRS405-PSGO1-Sgo1 mini-NLS-TAP | *MATa ade2-1 can1-100 his3-11 leu2-3,112 trp1-1 ura3-1 SGO1::hphNT1 tRTS1::eGFP-HIS3 tSPC29-RFP-natNT2 sgo1-mini-SV40NLS-TAP-LEU2* | This work |
| YZ1691 | W303 sgo1∆ Spc29-RFP pRS405-PSGO1-Sgo1 mini-NLS-GFP | *MATa ade2-1 can1-100 his3-11 leu2-3,112 trp1-1 ura3-1 SGO1::hphNT1 tSPC29::RFP-kanMX sgo1-mini-SV40NLS-GFP-LEU2* | This work |
| YZ1693 | W303 sgo1∆ pRS405-PSGO1-Sgo1 S148 A S151A-TAP pRS406-PGAL1-cik1-CC-TAP | *MATa ade2-1 can1-100 his3-11 leu2-3,112 trp1-1 ura3-1 SGO1::hphNT1 sgo1-S148A-S151A-TAP-LEU2 pGAL1-cik1-cc-TAP-URA3* | This work |
| YZ1697 | W303 sgo1∆ Rts1-eGFP Spc29-RFP pRS405-PSGO1-Sgo1 S151E-TAP | *MATa ade2-1 can1-100 his3-11 leu2-3,112 trp1-1 ura3-1 SGO1::hphNT1 tRTS1::eGFP-HIS3 tSPC29-RFP-natNT2 sgo1-S151E-TAP-LEU2* | This work |
| YZ1698 | W303 sgo1∆ Rts1-eGFP Spc29-RFP pRS405-PSGO1-Sgo1 S148E-TAP | *MATa ade2-1 can1-100 his3-11 leu2-3,112 trp1-1 ura3-1 SGO1::hphNT1 tRTS1::eGFP-HIS3 tSPC29-RFP-natNT2 sgo1-S148E-TAP-LEU2* | This work |
| YZ1699 | W303 sgo1∆ Rts1-eGFP Spc29-RFP pRS405-PSGO1-Sgo1 T159E-TAP | *MATa ade2-1 can1-100 his3-11 leu2-3,112 trp1-1 ura3-1 SGO1::hphNT1 tRTS1::eGFP-HIS3 tSPC29-RFP-natNT2 sgo1-T159E-TAP-LEU2* | This work |
| YZ1700 | W303 sgo1∆ Spc29-RFP pRS405-PSGO1-Sgo1 S151E-GFP | *MATa ade2-1 can1-100 his3-11 leu2-3,112 trp1-1 ura3-1 SGO1::hphNT1 tSPC29::RFP-kanMX sgo1-S151E-GFP-LEU2* | This work |
| YZ1701 | W303 sgo1∆ Spc29-RFP pRS405-PSGO1-Sgo1 S148E-GFP | *MATa ade2-1 can1-100 his3-11 leu2-3,112 trp1-1 ura3-1 SGO1::hphNT1 tSPC29::RFP-kanMX sgo1-S148E-GFP-LEU2* | This work |
| YZ1702 | W303 sgo1∆ Spc29-RFP pRS405-PSGO1-Sgo1 T159E-GFP | *MATa ade2-1 can1-100 his3-11 leu2-3,112 trp1-1 ura3-1 SGO1::hphNT1 tSPC29::RFP-kanMX sgo1-T159E-GFP-LEU2* | This work |
| YZ1706 | pRS405-SGO1pr_SGO1wt-TAP | *MATa ade2-1 can1-100 his3-11 leu2-3,112 trp1-1 ura3-1 SGO1::hphNT1 sgo1wt-TAP-LEU2* | This work |
| YZ1707 | S288C sgo1∆  pRS405-SGO1pr_SGO1wt-TAP | *MATalpha his3∆ leu2∆ ura3∆ met15∆ SGO1::hphNT2 sgo1wt-TAP-LEU2* | This work |
| YZ1708 | S288C sgo1∆  pRS405_PSGO1_Sgo1 S151A_TAP | *MATalpha his3∆ leu2∆ ura3∆ met15∆ SGO1::hphNT2 sgo1- -S151A -TAP-LEU2* | This work |
| YZ1709 | S288C sgo1∆  pRS405_*pSGO1_sgo1* S148A S151A_TAP | *MATalpha his3∆ leu2∆ ura3∆ met15∆ SGO1::hphNT2 sgo1-S148A-S151A-TAP-LEU2* | This work |
| YZ1710 | S288C sgo1∆  pRS405_*pSGO1_sgo1* S148A S151A T159A_TAP | *MATalpha his3∆ leu2∆ ura3∆ met15∆ SGO1::hphNT2 sgo1-S148A-S151A-T159A-TAP-LEUa* | This work |
| YZ1722 | W303 sgo1∆ Spc29-RFP pRS405_PSGO1_Sgo1 mini_NLS_GFP pRS406-PGAL1-cik1-CC-TAP | *MATa ade2-1 can1-100 his3-11 leu2-3,112 trp1-1 ura3-1 SGO1::hphNT1 tSPC29::RFP-kanMX sgo1-mini-SV40NLS-GFP-LEU2 pGAL1-cik1-cc-TAP-URA3* | This work |
| YZ1723 | W303 sgo1Δ Spc29-RFP pRS405_PSGO_Sgo mini N51I_NLS_GFP | *MATa ade2-1 can1-100 his3-11 leu2-3,112 trp1-1 ura3-1 SGO1::hphNT1 tSPC29::RFP-kanMX PSGO_Sgo mini N51I_NLS_GFP-LEU2* | This work |
| YZ1724 | W303 *sgo1Δ*  Spc29-RFP pRS405-pSGO-Sgo mini T379D-NLS-GFP | *MATa ade2-1 can1-100 his3-11 leu2-3,112 trp1-1 ura3-1 SGO1::hphNT1 tSPC29::RFP-kanMX pSGO_Sgo mini T379D_NLS_GFP-LEU2* | This work |
