# Supplementary material for: Phospho-regulation of the Shugoshin - Condensin interaction at the centromere in budding yeast
Source: PLoS Genet. 2020 Aug 18;16(8):e1008569. doi: 10.1371/journal.pgen.1008569 (PMC7454948; doi:10.1371/journal.pgen.1008569)
Supplement: S2 Table — (DOCX) [file pgen.1008569.s006.docx]

**Supplementary table 2 List of plasmids created and used in this work**

| **Plasmid** | **Name** | **Markers** |
| --- | --- | --- |
| 14 | pGEX-2TK-*SGO1* | Ampicillin |
| 18 | pGEX-4T1-*hMps1* | Ampicillin |
| 19 | pGEX-4T1-*hMps1 KD* | Ampicillin |
| 23 | pGEX-2TK-*yMps1* | Ampicillin |
| 124 | pRS405-*pSGO1-SGO1*-TAP | Ampicillin, LEU2 |
| 127 | pRS405- *pSGO1-sgo1-T379D*-TAP | Ampicillin, LEU2 |
| 129 | pRS405- *pSGO1-sgo1-T379D*-eGFP | Ampicillin, LEU2 |
| 167 | pRS405- *pSGO1-sgo1-N51I*-TAP | Ampicillin, LEU2 |
| 333 | pRS405-*pSGO1-sgo1-Δ101-310aa*-TAP | Ampicillin, LEU2 |
| 346 | pRS405-pSGO1-sgo1-*Δ101-310aa* -eGFP | Ampicillin, LEU2 |
| 347 | pRS405-*pSGO1-SGO1*-eGFP | Ampicillin, LEU2 |
| BZ427 | pRS406-*pGAL1*-*cik1-cc*-TAP | Ampicillin,URA3 |
| BZ542 | pRS405-*pSGO1- sgo1*-*Δ201-310aa*-eGFP | Ampicillin, LEU2 |
| BZ557 | pGEX-2TK-*sgo1-141-163aa* | Ampicillin |
| BZ558 | pGEX-2TK-*sgo1 1-163aa* | Ampicillin |
| BZ559 | pGEX-2TK-*sgo1* *101-200aa* | Ampicillin |
| BZ560 | pRS405-*pSGO1-sgo1*-*Δ101-250aa*-TAP | Ampicillin, LEU2 |
| BZ561 | pRS405-*pSGO1-sgo1*-*Δ101-180aa*-TAP | Ampicillin, LEU2 |
| BZ564 | pRS405-*pSGO1-sgo1*-*Δ137-163aa*-TAP | Ampicillin, LEU2 |
| BZ566 | pGEX-2TK- *sgo1-N51I* | Ampicillin |
| BZ567 | pGEX-2TK- *sgo1*-*Δ137-163aa* | Ampicillin |
| BZ571 | pRS405-*pSGO1-sgo1*-*13A*-TAP | Ampicillin, LEU2 |
| BZ572 | pRS405-*pSGO1-sgo1*-*13A*-eGFP | Ampicillin, LEU2 |
| BZ582 | pRS405-*pSGO1-sgo1 mini-NLS*-TAP | Ampicillin, LEU2 |
| BZ583 | pRS405-*pSGO1-sgo1 mini-NLS*-eGFP | Ampicillin, LEU2 |
| BZ586 | pRS405-*pSGO1-sgo1*-*S151E*-TAP | Ampicillin, LEU2 |
| BZ587 | pRS405-*pSGO1-sgo1*-*S151E*-eGFP | Ampicillin, LEU2 |
| BZ588 | pRS405-*pSGO1-sgo1*-*S148E*-TAP | Ampicillin, LEU2 |
| BZ589 | pRS405-*pSGO1-sgo1*-*S148E*-eGFP | Ampicillin, LEU2 |
| BZ590 | pRS405-*pSGO1-sgo1*-*T159E*-TAP | Ampicillin, LEU2 |
| BZ591 | pRS405-*pSGO1-sgo1*-*T159E*-eGFP | Ampicillin, LEU2 |
| BZ592 | pRS405-*pSGO1- sgo1 mini-N51I-NLS*-eGFP | Ampicillin, LEU2 |
| BZ593 | pRS405-*pSGO1-sgo1 mini-T379D-NLS*-eGFP | Ampicillin, LEU2 |
| BZ594 | pGEX-2TK-*sgo1 mini* | Ampicillin |
| BZ596 | pRS405-*pSGO1-sgo1*-*S148E, S151E, T159E*-TAP | Ampicillin, LEU2 |
| BZ599 | pRS405-*pSGO1-sgo1*-*13E*-TAP | Ampicillin, LEU2 |
| BZ600 | pRS405-*pSGO1-sgo1*-*13E*-eGFP | Ampicillin, LEU2 |
| BZ610 | pRS405-*pSGO1-sgo1-Δ141-167aa*-TAP | Ampicillin, LEU2 |
| BZ611 | pRS405-*pSGO1-sgo1-Δ101-200aa*-TAP | Ampicillin, LEU2 |
| BZ612 | pRS405-*pSGO1-sgo1-Δ201-310aa*-TAP | Ampicillin, LEU2 |
| BZ616 | pRS405-*pSGO1-sgo1-S148A, S151A*-TAP | Ampicillin, LEU2 |
| BZ617 | pRS405-*pSGO1-sgo1-S148A, S151A, T159A*-TAP | Ampicillin, LEU2 |
| BZ618 | pRS405-*pSGO1-sgo1-S151A*-TAP | Ampicillin, LEU2 |
| BZ620 | pRS405-*pSGO1-sgo1-T159A*-TAP | Ampicillin, LEU2 |
| BZ621 | pRS405-*pSGO1-sgo1-S151A*-TAP | Ampicillin, LEU2 |
| BZ622 | pRS405-*pSGO1-sgo1-T159A*-eGFP | Ampicillin, LEU2 |
| BZ623 | pRS405-*pSGO1-sgo1-3A*-TAP | Ampicillin, LEU2 |
| BZ624 | pRS405-*pSGO1-sgo1-3E*-TAP | Ampicillin, LEU2 |
| BZ625 | pRS405-*pSGO1-sgo1-3A*-eGFP | Ampicillin, LEU2 |
| BZ626 | pRS405-*pSGO1-sgo1-3E*-eGFP | Ampicillin, LEU2 |
| BZ627 | pRS405-*pSGO1-sgo1-T159F*-TAP | Ampicillin, LEU2 |
| BZ628 | pRS405-*pSGO1-sgo1-T159Q*-TAP | Ampicillin, LEU2 |
| BZ629 | pRS405-*pSGO1-sgo1-T159K*-TAP | Ampicillin, LEU2 |
